# Anti-CV2 Antibody-Positive Sensorimotor Polyneuropathy Following Bacillus Calmette-Guérin Intravesical Infusion Therapy

**DOI:** 10.7759/cureus.67686

**Published:** 2024-08-24

**Authors:** Takuya Saito, Yoshiyuki Kondo, Kosuke Uchida, Keishiro Sato, Tsuyoshi Uchiyama

**Affiliations:** 1 Department of Neurology, Seirei Hamamatsu General Hospital, Hamamatsu, JPN; 2 Department of Urology, Seirei Hamamatsu General Hospital, Hamamatsu, JPN

**Keywords:** neurology, immunotherapy, urinary bladder neoplasms, vaccination, peripheral neuropathies

## Abstract

Bacillus Calmette-Guérin (BCG) intravesical infusion therapy is widely used to control recurrence after transurethral resection of bladder tumors. Herein, we report a case of polyneuropathy with transiently positive onconeural antibodies after BCG bladder infusion therapy. A man in his 70s presented with upper and lower extremity weakness 11 weeks after BCG intravesical infusion therapy, a postoperative therapy for superficial bladder cancer. Nerve conduction studies revealed findings that were consistent with demyelinating sensorimotor polyneuropathy. Anti-CV2 antibody was positive; however, contrast-enhanced computed tomography and positron emission tomography revealed no malignancy. The patient’s symptoms improved with immunoglobulin therapy. Contrast-enhanced computed tomography showed no malignancy, and the anti-CV-2 antibody test result was negative six months after discharge. The immune response to BCG bladder infusion therapy may have caused the transient CV2 antibody positivity and polyneuropathy. The possibility of transiently positive onconeural antibodies after BCG intravesical infusion therapy should be considered.

## Introduction

Bacillus Calmette-Guérin (BCG) intravesical infusion therapy is widely used to treat residual tumors and control recurrence after transurethral resection of bladder tumors [1]. BCG intravesical infusion therapy is known to cause a variety of local and systemic complications, and although rare, neuropathy has been reported [2]. Neurological syndromes caused by BCG bladder infusion therapy are assumed to be immune-based pathologies [3-5], and paraneoplastic neurologic syndromes (PNS) have been reported in a small number of cases [6]. Testing for nerve onconeural antibodies is important for the diagnosis of PNS, and various onconeural antibodies have become available in recent years [7]. However, onconeural antibody tests are often false positives [8]. Herein, we report a case of polyneuropathy with transiently positive onconeural antibodies after BCG bladder infusion therapy.

## Case presentation

**Figure 1:**
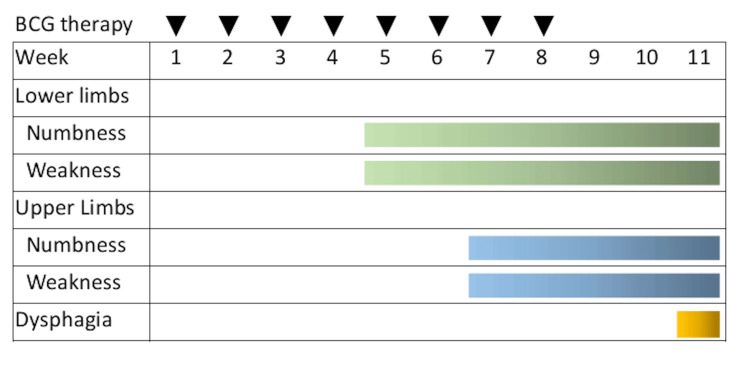
Timeline of the patient’s clinical course

A man in his 70s with no significant medical history other than heavy alcohol consumption (60 g of ethanol per day) was diagnosed with bladder cancer following hematuria. He underwent transurethral resection, and histological examination revealed a papillary type of high-grade urothelial carcinoma with a tumor, node, metastasis (TNM) stage of pT1N0M0, concomitant carcinoma in situ (CIS). Lymphovascular invasion was not seen in the tissue examined. Weekly BCG intravesical infusion therapy (BCG Tokyo-172 strain) was started four weeks after surgery. He developed numbness and weakness in the distal parts of both lower limbs and lower back pain five weeks after starting BCG intravesical infusion therapy. Seven weeks later, the patient developed numbness and weakness in the distal parts of both the upper limbs. Eight sessions of BCG intravesical infusion therapy were administered. His symptoms progressed 11 weeks later; he was unable to walk and had dysphagia. He visited our hospital (Figure 1).

**Figure 2:**
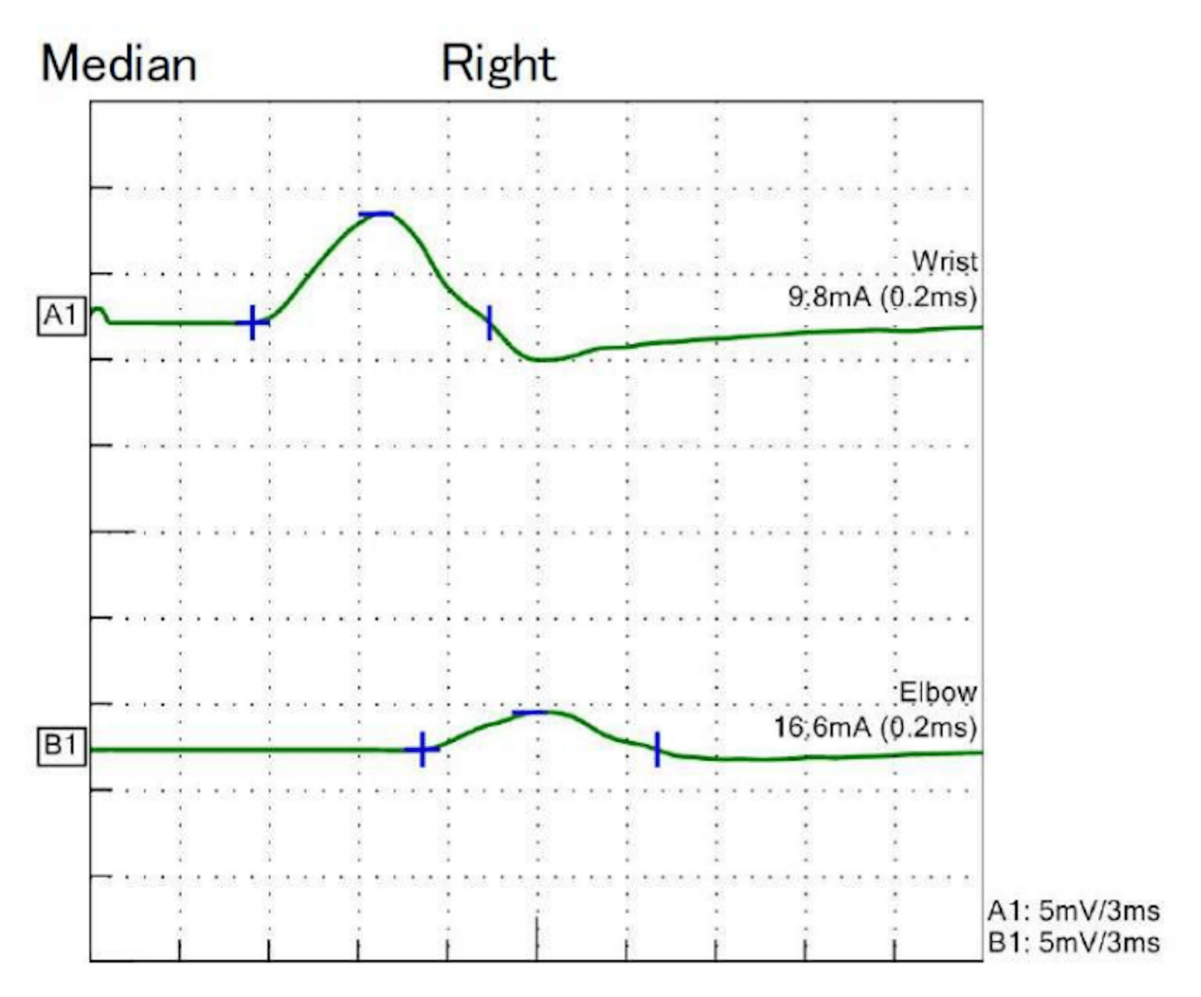
Right median nerve motor conduction studies

His blood pressure was 156/105 mmHg, and his pulse rate was 94 beats/minute. His pupils were 3 mm bilaterally, his light reflex and eye movements were normal, and his vision was normal. His pharyngeal function and gag reflex were decreased. Facial muscle strength, soft palate movement, and tongue movement were normal. In the manual muscle test, the trapezius muscle was bilaterally grade 5, the other upper extremity muscles were bilaterally grade 4, and the lower extremity muscles were all bilaterally grade 3. Pain, vibration, and position sensations were mildly decreased in the extremities. Paresthesia was present in the extremities. Deep tendon reflexes and abnormal reflexes were absent in the extremities. Cerebellar signs were absent. Nerve conduction studies (NCS) showed decreased sensory nerve action potential, and polyneuropathy was diagnosed. Blood tests showed no significant results other than a positive anti-CV2 antibody (Table 1). Cerebrospinal fluid (CSF) examination showed elevated CSF protein (Table 1). Contrast-enhanced computed tomography and positron emission tomography (PET) showed no malignancy, such as small cell carcinoma of the lung. Magnetic resonance imaging showed no nerve root enlargement, contrast effect, or contrast effect on the muscle. Abdominal wall fat biopsy revealed no amyloids. Urinary cytology and cystoscopy revealed no recurrence of bladder cancer. The NCS were re-evaluated three weeks after admission. A conduction block in the right median nerve; prolonged distal latency in the right median, right ulnar, right tibial, and right sural nerves; prolonged F-wave latency in the right median nerve; reduction of motor conduction velocity in the right median and tibial nerves; reduction of sensory action potential in the right median, right ulnar, and right sural nerves; and reduction of sensory conduction velocity in the right median, right ulnar, and right sural nerves were observed (Figure 2, Table 2). All findings were consistent with demyelinating sensorimotor neuropathy in the European Academy of Neurology/Peripheral Nerve Society guideline of chronic inflammatory demyelinating polyradiculoneuropathy [9].

**Table 1:** Laboratory test results AFP, alpha-fetoprotein; CA19-9, carbohydrate antigen 19-9; CEA, carcinoembryonic antigen; CSF, cerebrospinal fluid; HbA1c, glycated hemoglobin; HBs, hepatitis B surface; MPO-ANCA, myeloperoxidase-specific antineutrophil cytoplasmic antibody; PR3-ANCA, proteinase 3-specific antineutrophil cytoplasmic antibody; ProGRP, pro-gastrin-releasing peptide; PSA, prostate-specific antigen; sIL-2R, soluble interleukin-2 receptor; SS-A, Sjogren's Syndrome-A; SS-B, Sjogren's Syndrome-B; T-SPOT, T-cell spot test for tuberculosis

Laboratory test	Value	Reference range
HbA1c (%)	5.5	4.9-6.0
Vitamin B1 (ng/dL)	55	24-66
Vitamin B12 (pg/dL)	374	233-914
Folic acid (ng/dL)	4.2	2.4-10.0
Copper levels (µg/dL)	110	68-128
Free T4 (ng/dL)	0.85	0.7-1.5
HBs antigen	Negative	Negative
Anti-HCV antibody	Negative	Negative
Anti-HIV antibody	Negative	Negative
T-SPOT	Negative	Negative
Anti-nuclear antibody	Negative	Negative
Anti-ds-DNA antibody (IU/mL)	<10	<12
PR3-ANCA (U/mL)	<1.0	<3.5
MPO-ANCA (U/mL)	<1.0	<3.5
Anti-GM1 antibody	Negative	Negative
Anti-GQ1b antibody	Negative	Negative
Anti-SS-A antibody (U/mL)	<1.0	<10
Anti-SS-B antibody (U/mL)	<1.0	<10
sIL-2R (U/mL)	247	157-474
Anti-Hu antibody	Negative	Negative
Anti-Yo antibody	Negative	Negative
Anti-CV2 antibody	Positive	Negative
PSA (ng/mL)	0.96	<4.0
CEA (ng/mL)	1.9	0-5
AFP (ng/mL)	3.6	0-8.8
CA19-9 (U/mL)	5.4	0-37
ProGRP (pg/mL)	33	<81.0
CSF protein (mg/dL)	64	15-45
CSF cell count (/µL)	2	<5

**Table 2:** Nerve conduction studies results *Compound muscle action potential (mV); sensory action potential (µV)

Nerve	Distal Latency	Amplitude*	Velocity	F-latency
	(ms)	Distal/proximal	(m/s)	(ms)
Motor				
Median	5.5	6.3/2.2	36.8	37
Ulnar	4	5.4/4.7	50.9	32.7
Tibial	6.2	7.1/6.0	35.5	54.1
Sensory				
Median	3.9	1.7/not evoked	40.6	NA
Ulnar	3	3.3/not evoked	42.8	NA
Sural	3.8	3.8/not evoked	40.6	NA

Systemic management, including administration of vitamin B1, was performed after admission. Immunoglobulin therapy (0.4 g/kg/day for five days) was initiated seven days after admission because limb muscle weakness continued to progress. The patient’s symptoms improved gradually after four days of immunoglobulin therapy initiation. The patient was discharged after rehabilitation with no sequelae or disease re-exacerbation. NCS findings improved after discharge. The anti-CV-2 antibody became negative six months after discharge. Contrast-enhanced computed tomography revealed no malignancy one year after discharge. Urinary cytology and cystoscopy revealed no recurrence of bladder cancer one year after discharge. We hypothesized that the immunological mechanism of BCG bladder infusion therapy caused polyneuropathy and transiently positive anti-CV2 antibodies.

## Discussion

This is a case of anti-CV2 antibody-positive sensorimotor neuropathy after BCG intravesical infusion therapy. Neurological symptoms worsened over a three-month period and improved rapidly after immunoglobulin therapy. The patient was transiently positive for anti-CV2 antibodies but had no malignancy.

Anti-CV2 antibodies have been reported to be strongly associated with PNS, especially in small-cell carcinoma of the lung. However, anti-CV2 antibody-positive neuropathy associated with bladder cancer has not been reported. The sensorimotor neuropathy, in this case, was considered to be a neuronopathy with additional sensory and motor nerve involvement and was a high-risk neurologic phenotype of PNS.

A diagnosis of PNS with anti-CV2 antibodies should be made, even if no malignancy is found [7]. The neurological symptoms may present earlier than the detection of malignancy on imaging studies, and continued malignancy evaluation for two years is recommended [7]. In contrast, Ruiz-Garcia et al. reported that only half of anti-CV2 antibody-positive neurological syndromes were diagnosed as PNS, and anti-CV2 antibody false-positives were common [8]. If onconeural antibodies are positive in serum, repeat CSF testing, repeat testing in research laboratories, and confirmation by brain immunohistochemistry are recommended [7]. However, none of these are common and could not be evaluated in this case.

Zhu et al. [6] reported that the presentation of autoimmune antigens owing to BCG intravesical infusion therapy amplified the autoimmune response and promoted the appearance of neurological signs. They hypothesized that the immune system responds to antigen presentation by biologics, prompting the release of cytokines and chemokines that promote major histocompatibility complex (MHC) class I upregulation on neural cells. Then, neural cells are attacked by activated cluster of differentiation 8 (CD8)-positive T-cells due to pre-existing anti-tumor response and MHC, resulting in neurological symptoms [6]. In this case, the immune response by the remaining CIS may have caused the activation of CD8-positive T-cells. Neurologic symptoms occurred within one week to several months of BCG administration in previous reports [3,5,6], which is consistent with this case. The neurological symptoms worsened with continued BCG bladder infusion therapy in this case, which has not been reported before. An immune mechanism due to BCG administration is strongly suspected based on the course of the disease, which improved with immunotherapy, although the detailed pathomechanism is unknown.

## Conclusions

If polyneuropathy develops after BCG intravesical infusion therapy, the possibility of PNS should be considered, and a close examination should be performed. Moreover, the possibility of transiently positive onconeural antibodies after BCG intravesical infusion therapy should be considered.

## References

[1] Babjuk M, Böhle A, Burger M (2017). EAU guidelines on non-muscle-invasive urothelial carcinoma of the bladder: update 2016. Eur Urol.

[2] Pérez-Jacoiste Asín MA, Fernández-Ruiz M, López-Medrano F (2014). Bacillus Calmette-Guérin (BCG) infection following intravesical BCG administration as adjunctive therapy for bladder cancer: incidence, risk factors, and outcome in a single-institution series and review of the literature. Medicine (Baltimore).

[3] Webb K, Venkatesan P (2018). Guillain Barré syndrome associated with bladder instillation of Bacille Calmette Guérin (BCG). JMM Case Rep.

[4] Becker A, Grunwald IQ, Unger MM (2020). Progressive cerebral small vessel disease caused by an autoimmune response to intravesical Bacille-Calmette-Guérin treatment. Front Neurol.

[5] Katznelson D, Gross S, Sack J (1982). Polyneuritis following BCG re-vaccination. Postgrad Med J.

[6] Zhu M, Ma Y, Zekeridou A, Lennon VA (2020). Case report: innate immune system challenge unleashes paraneoplastic neurological autoimmunity. Front Neurol.

[7] Graus F, Vogrig A, Muñiz-Castrillo S (2021). Updated diagnostic criteria for paraneoplastic neurologic syndromes. Neurol Neuroimmunol Neuroinflamm.

[8] Ruiz-García R, Martínez-Hernández E, Saiz A, Dalmau J, Graus F (2020). The diagnostic value of onconeural antibodies depends on how they are tested. Front Immunol.

[9] Van den Bergh PY, van Doorn PA, Hadden RD (2021). European Academy of Neurology/Peripheral Nerve Society guideline on diagnosis and treatment of chronic inflammatory demyelinating polyradiculoneuropathy: report of a joint task force-second revision. Eur J Neurol.

